# *Burkholderia cepacia* lipase immobilized on heterofunctional magnetic nanoparticles and its application in biodiesel synthesis

**DOI:** 10.1038/s41598-017-16626-5

**Published:** 2017-11-28

**Authors:** Kai Li, Yanli Fan, Yaojia He, Leping Zeng, Xiaotao Han, Yunjun Yan

**Affiliations:** 1 Key Laboratory of Molecular Biophysics of the Ministry of Education, College of Life Science and Technology, Huazhong University of Science and Technology, Wuhan, 430074 China; 2 Wuhan National High Magnetic Field Center, Huazhong University of Science and Technology, Wuhan, 430074 P.R. China

## Abstract

Biodiesel production using immobilized lipase as a biocatalyst is a promising process. The performance of immobilized lipase is mainly determined by supporting materials and immobilization method. To avoid the shortcomings of adsorption and covalent bonding methods, in this study, we developed a novel heterofunctional carrier of being strengthened anion exchange and weakened covalent binding to avoid activity loss and improve operational stability of the immobilized lipase. 2,3-epoxypropyltrimethylammonium chloride with epoxy and quaternary ammonium group and glutaraldehyde were grafted onto aminated magnetic nanoparticles (AMNPs) to generate a new matrix, named GEAMNP. Then *Burkholderia cepacia* lipase (BCL) was immobilized on GEAMNP via anion exchange and covalent bonding. The transesterification between soybean oil and methanol was used to test the activities. Activity recovery of the immobilized BCL was up to 147.4% and the corresponding transesterification activity was 1.5-fold than that of BCL powder. The immobilized lipase was further used for biodiesel production to confirm its feasibility. The fatty acid methyl esters conversion yield could reach 96.8% in the first 12 h. Furthermore, the immobilized lipase, BCL-GEAMNP showed markedly improved operational stability, better reusability and higher esters than BCL-GAMNP, where MNPs were only modified with (3-aminopropyl) triethoxysilane and glutaraldehyde.

## Introduction

The sustainable development of mankind requires non-toxic, biodegradable and renewable fuel and energy^1,2^. Biodiesel is a renewable and clean energy. It is produced via transesterification of oils or fats and short chain alcohols catalyzed by chemicals (such as acid/base) or lipases^3^. Lipase has many advantages such as mild operating conditions, environmentally friendly, adaptable to different biodiesel feedstock, and having an easy downstream procedure^4^. However, there are two main disadvantages in lipase-catalyzed biodiesel production, high price and easy inactivation of lipases. Immobilization of lipase is a potential strategy for solving these problems. Immobilization enhances their storage and operational stability^5–10^, recyclability^5,8,10^, activity and substrate specificity^8,9,11,12^, which makes lipase possible for enzyme-based industrial applications^8,11^. So, in this study, suitable immobilization systems, methods and carriers need to be explored.

Many studies on lipase immobilization have been reported. The most common immobilization technologies are cross-linking, entrapment and binding to carriers (including physical adsorption, ion exchange, covalent binding and complexation)^13^. One of the immobilization methods called multipoint covalent bonding can increase the stability of monomeric enzyme and prevent enzyme conformational changes^9,12^. But sometimes covalent coupling often cause enzyme inactivation^14^. In addition to these technologies, heterofunctional supports have recently gained increasing attention as it can combine the advantages of different methods and achieve an improved and controlled immobilization^15^. There are some examples about heterofunctional immobilization. Cao *et al*. reported the use of a novel low-cost material, magnetic cellulose nanocrystals (MCNCs), and combined adsorption (lipase–carrier) and cross-linking (lipase–lipase) together. For heterofunctional immobilization, physical adsorption together with ion exchange and physical adsorption coupling with covalent binding have been reported^16^. Zheng *et al*. immobilized *Candida rugosa* lipase on a novel composite material, hyper-cross-linked polymer-coated silica (HPCS) particles, via a hydrophobic and weak cation exchange interaction^17^. Gao *et al*. measured the isotherm adsorption behaviors and the isocratic retention factors of bovine serum albumin on Streamline Direct HST which is a new type of heterofunctional adsorbent with a hydrophobic and cation exchange ligand^18^. Boros *et al*. developed an ideal support for both adsorption and covalent binding for lipase B from *Candida antarctica* ^19^. Arroyo *et al*. proved that the thermal stability of lipase B from *Candida antarctica* immobilized on amino-silica supports was better than that prepared by physical adsorption only^20^. Another interesting heterofunctional support prepared with physical adsorption and covalent binding has also been reported by Wang *et al*. via using the block copolymer grafted Fe_3_O_4_ particles^21^. However, from the above analysis, in order to achieve one step immobilization, the hybrid strategy of strengthened anion exchange coupling with weakened multi-point covalent binding can be used to avoid activity loss and improve operational stability of the immobilized enzyme. Thus, in this study, we chose quaternary ammonium groups and aldehyde groups to generate ion exchange and predominantly employed lysine residues on the BCL molecule to realize covalent binding to enhance the effect of immobilization.

Glutaraldehyde was one of the most widely used reagents covalently immobilize enzymes in carriers^22^ and was used to activate the aminated carriers in this study. It reacted predominantly with the ε-amino groups of lysine. Other groups like thiols, phenols, and imidazoles can also react with it^23,24^. There are two different immobilization strategies: one is to adsorb the enzyme on aminated carriers by ionic exchange followed by treating this composite with glutaraldehyde under mild conditions; The other is to use a pre-activated support to immobilize the target protein^22^. Compared with the first strategy, the second one can avoid inter and intramolecular protein crosslinking. In addition to the covalent attachment for lipase immobilization, another advantage of glutaraldehyde is the hydrophobic nature which permit the adsorption of lipases via the so-called interfacial activation^22^. A polypeptide chain called lid that surrounded the active center of the lipase. When the lipase contacts with some hydrophobic groups or hydrophobic substrates, the lid moves followed by the active center exposes and changes from a closed/inactive form to an opened/active form. This is the interfacial activation^22^. When the carrier is modified with glutaraldehyde, interfacial activation versus the support may be a driving force of the immobilization just like ion exchange and covalent bonding. Some of the researchers combine interfacial activation with adsorption or covalent attachment. Abaházi *et al*. used several additives such as PEGs, oleic acid and polyvinylalcohol to enhance the activity of BCL and immobilized BCL by adsorption followed by cross-linking on mesoporous silica gel^25^. Enzyme immobilized on hydrophobic supports via interfacial activation has also been reported by many researchers^15,26,27^.

In addition, various materials used in enzyme immobilization have been reported in the literature. Synthetic environmentally sensitive carriers such as thermosensitive carriers^28,29^, pH sensitive hydrogel^30^, magnetic response carriers (including shell/core structure^31^, core/shell structure^32^ and heterofunctional structure^33^) and multi-responsive carriers^34^, can respond to environmental stimulation factors. These factors include temperature, light, magnetic field, electric field, pH value of buffer, solvent, reactant, ion or stress. So far, studies on environmental stimulation factors have gained wide attention in biomedicine (i.e. enzyme and cell immobilization), drug delivery and chemical engineering. These materials can change their natural properties and show special advantages when they respond to the environmental factors. Of the environmental factors, temperature and pH value are easier to control, but also have some limitations when used as control signals in enzyme immobilization. For example, some materials can change pore sizes at high temperature or in acidic/alkaline conditions which is good for increasing immobilization efficiency, however, enzyme will be inactivated under these extreme conditions. In comparison, magnetic nanoparticles have gained considerable attention in recent years as immobilized lipase with magnetic carriers is easily operated in separation and recycling procedures. Magnetic nanoparticles can also decrease mass transfer resistance in reactions and have a high enzyme loading owing to the high surface area of the carriers, but the cost of nanoparticles constrict their large scale application in industry^35^. In biodiesel production, compared with non-porous nanoparticles, porous supports have external diffusion problems and may accumulate glycerin or water and promote enzyme inactivation that cannot occur using nonporous materials. However, for nonporous nanoparticles, enzyme can only be immobilized on the external surface of the carrier. That can lead the enzyme to lose protection from interactions with hydrophobic interfaces. And some enzyme on the external surface can directly interact with other molecules in other particle which may lead to proteolysis^35^.

Therefore, in the present study, heterofunctional and magnetic carriers were employed to immobilize *Burkholderia cepacia* lipase (BCL), a widely applied enzyme in industries. The immobilized lipase, BCL-GEAMNP (magnetic nanoparticles grafted with quaternary ammonium groups and aldehyde groups), was successfully prepared and then used to catalyze transesterification for biodiesel production. In addition, the activity and operational stability of the immobilized lipase were compared with those immobilized lipases previously reported.

## Results

### Synthesis and characterization of the carriers (MNP, AMNP, EAMNP and GEAMNP) and immobilized lipase (BCL-GEAMNP)

**Figure 1:**
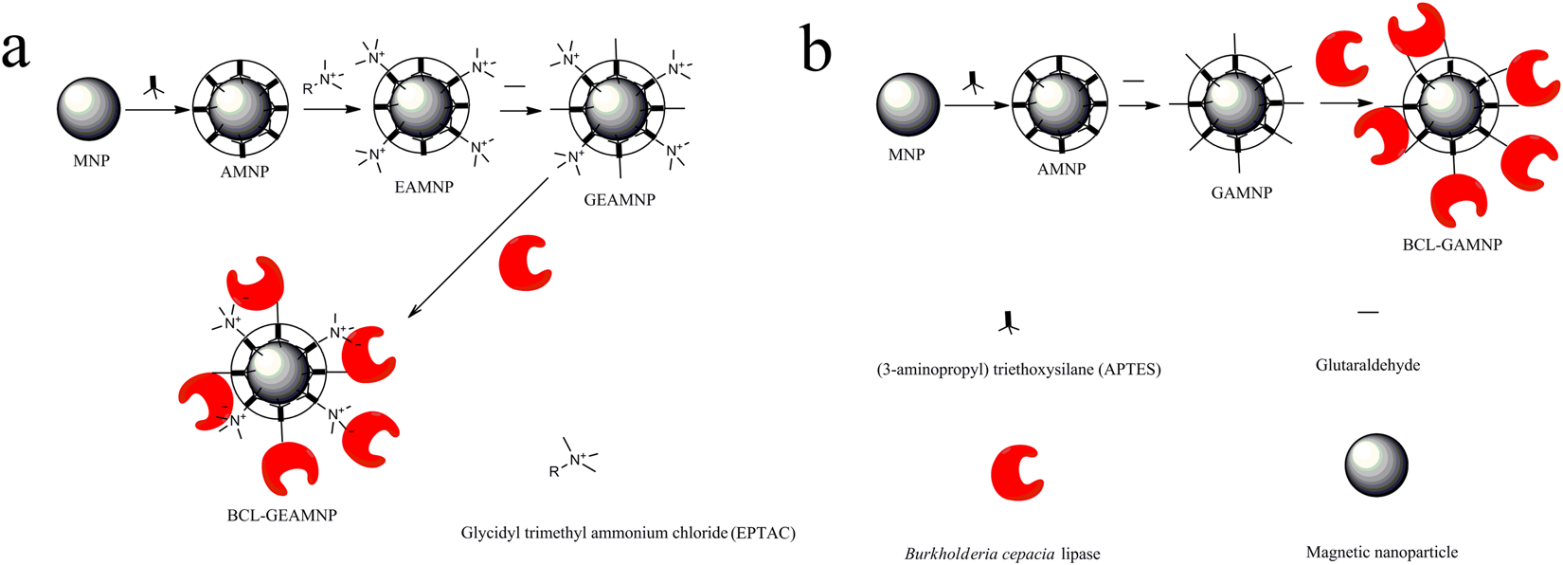
A scheme of GEAMNP (**a**) and GAMNP (**b**) synthesis for modification of MNP and BCL immobilization.

The synthesis strategy for magnetic nanoparticles (MNPs) modified with (3-aminopropyl) triethoxysilane (APTES), 2,3-epoxypropyltrimethylammonium chloride (EPTAC) and glutaraldehyde and the immobilized lipase, BCL-GEAMNP, are shown in Fig. 1. First, APTES was grafted onto the surface of MNPs to form amino-functionalized MNPs (AMNPs). Then, quaternary amine groups were grafted onto AMNPs through the interaction between the epoxy group of EPTAC and the amino group of AMNP (carrier named as EAMNP). Glutaraldehyde was thereafter grafted onto the carrier through the interaction between the aldehyde group and the remaining amino group of AMNP (carrier named as GEAMNP). Finally, BCL was coupled onto the modified carrier.

**Figure 2:**
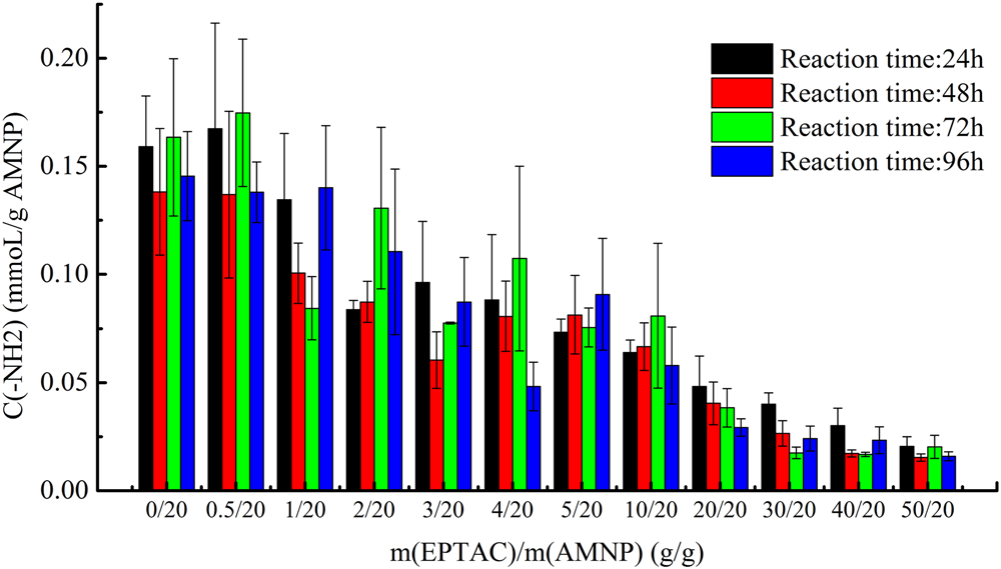
Effects of reaction time and mass ratio of EPTAC to AMNP on the molarity of the amino group remaining on the surface of the carriers.

**Figure 3:**
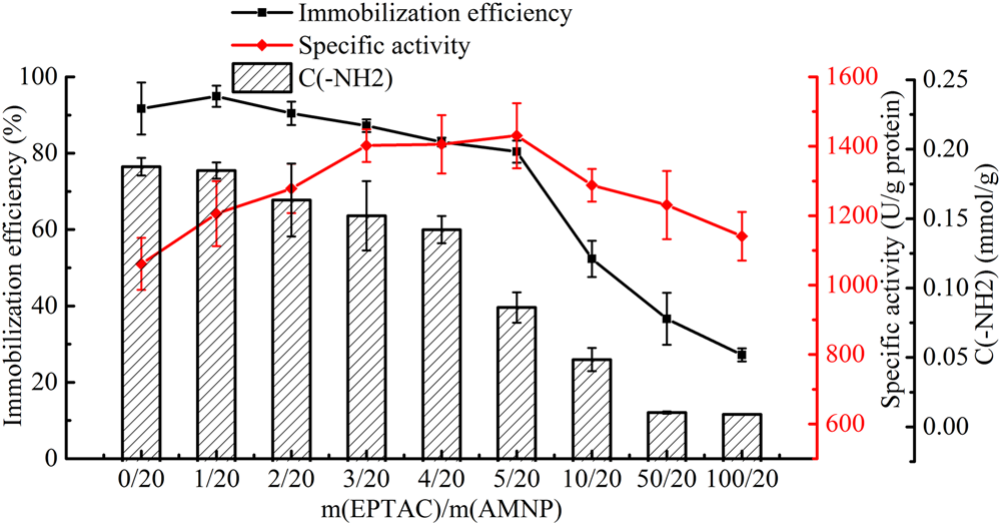
Effects of mass ratio of EPTAC to AMNP on the distribution of quaternary and primary amine groups on the carriers, and immobilization efficiency and specific activity of immobilized lipase.

As shown in Fig. 1, EPTAC and glutaraldehyde were on the same layer of the carrier, but they were grafted onto the carrier in first and second order. In order to test the effect of anion exchange and covalent binding on lipase immobilization, the appropriate proportion of the coupled EPTAC and the remaining amino group on AMNP required to be confirmed. As shown in Fig. 2, the initial amount of EPTAC added was the most relevant factor to the molarity of quaternary amine modified on the surface of the carrier. When the mass ratio of EPTAC to AMNP changed from 0/20 to 50/20, the EPTAC loading increased, whereas, the concentration of primary amine decreased. The reaction time (from 24 h to 96 h) had no significant effect on EPTAC loading. Thus, 24 h was chosen as the appropriate reaction time. Then, in order to compare the specific activities of the immobilized lipases with different proportions of quaternary amine groups and aldehyde groups, BCL was immobilized on different carriers as mentioned above. The content of the remaining amino group on the carriers, immobilization efficiencies and specific activities of the immobilized lipases are shown in Fig. 3. It can be seen that primary amine was increasingly replaced by the quaternary amine as more EPTAC was added into the system. Immobilization efficiency decreased gradually when the mass ratio of EPTAC to AMNP increased from 0/20 to 100/20. However, when the lipase content remained unchanged, the specific activity rose to the maximal value as the mass ratio was 5/20, and then decreased. Thus, the carrier with proper proportion of quaternary amine groups and aldehyde groups performs better than the one with aldehyde groups only. 5/20 was selected as the optimum mass ratio of EPTAC to AMNP, and this ratio was used in subsequent studies.

The modified carriers and immobilized lipase were investigated using X ray diffraction (XRD), Fourier transform infrared spectroscopy (FT-IR), X-ray photoelectron spectroscopy (XPS), transmission electron microscopy (TEM), atomic force microscopy (AFM) and confocal laser scanning microscopy (CLSM). A vibrating sample magnetometer (VSM) was used to test the magnetic variation after modification and immobilization.

**Figure 4:**
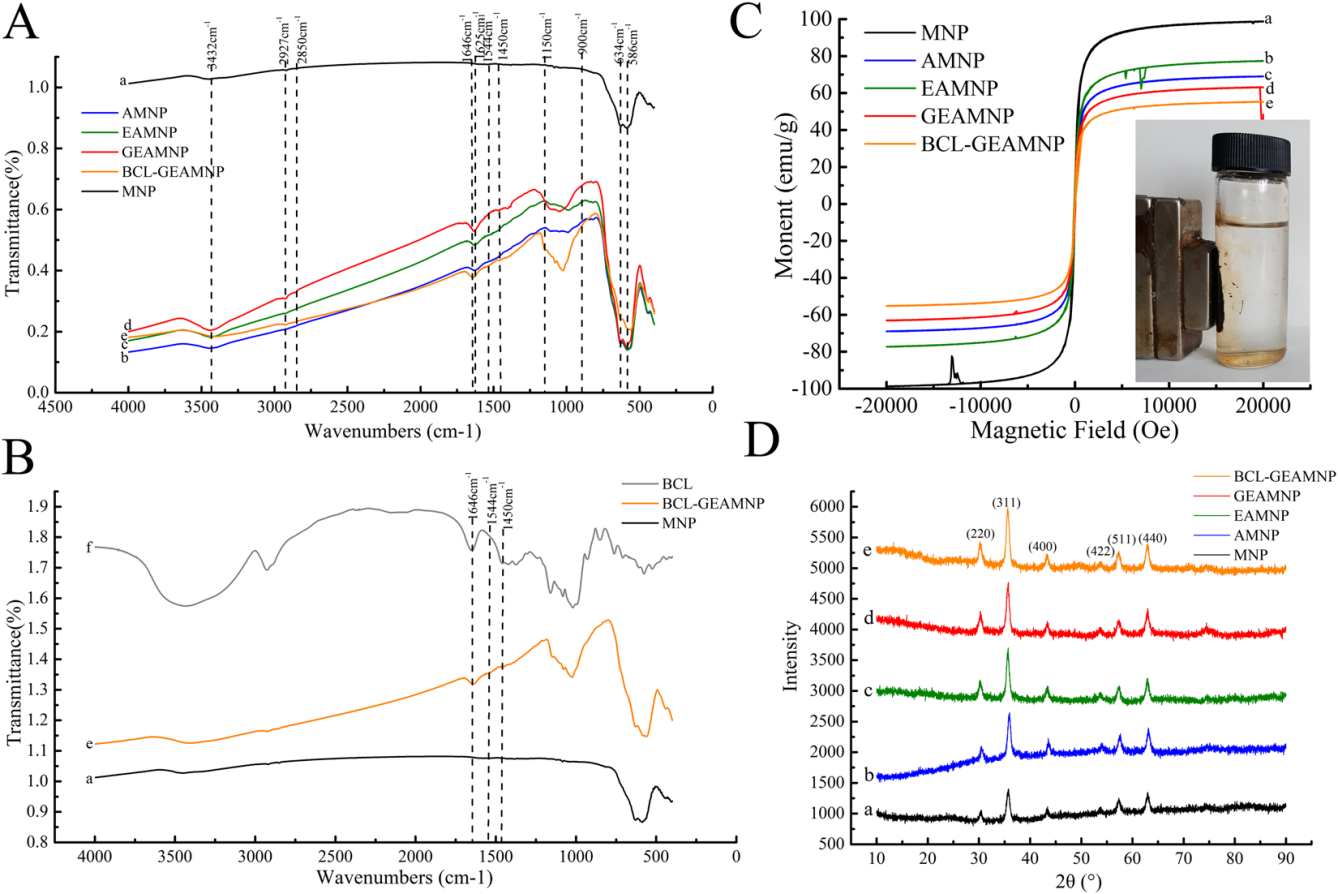
(**A**) Fourier transform infrared (FT-IR) spectra of samples: (a) MNP; (b) AMNP; (c) EAMNP; (d) GEAMNP and (e) BCL-GEAMNP. (**B**) FT-IR spectra of samples: (a) MNP; (e) BCL-GEAMNP and (f) BCL powder. (**C**) The hysteresis loops of MNP, AMNP, EAMNP, GEAMNP, and BCL-GEAMNP. (**D**) X Ray diffraction (XRD) patterns of samples: (a) MNP; (b) AMNP; (c) EAMNP; (d) GEAMNP and (e) BCL-GEAMNP.

The FT-IR spectra of MNP, AMNP, EAMNP, GEAMNP, and BCL-GEAMNP are shown in Fig. 4Aa–e. The strong absorption band around 586 and 634 cm^−1^ is ascribed to the Fe-O bond of Fe_3_O_4_^36,37^ (Fig. 4Aa–e). The C–H stretching vibrations of the grafted organic molecules (APTES, EPTAC, glutaraldehyde, and lipase) are observed at 2865 and 2925 cm^−1^ (Fig. 4Ab–e)^38^. The absorption band at 900–1150 cm^−1^ is attributed to the stretching vibration of the Si–O bond (Fig. 4Ab–e)^39^. Figure 4Ba,e,f represent the absorption bands of MNP, BCL-GEAMNP and BCL powder, respectively. Absorption bands at peak 1646 cm^−1^ and 1544 cm^−1^ are characteristics of amide (–CO–NH–) I and II bonds for lipase (Fig. 4Ae and Be)^40^. Bands at 1646 cm^−1^ can also be attributed to stretching vibration for imine group. However, there are also many interference peaks of organic compounds on the supports.

As shown in Fig. 4C, VSM measurements have proved that the saturation magnetization (MS) values of MNP, AMNP, EAMNP, GEAMNP and BCL-GEAMNP are 99 emu/g, 69 emu/g, 77 emu/g, 48 emu/g and 55 emu/g, respectively. The magnetization curves exhibit no hysteresis for all of the samples, indicating their superparamagnetic character. Thus, the samples can be rapid separated from the reaction mixture using a magnet in a very short time. Once the magnetic field has been removed, the immobilized enzyme can be easily dispersed in the reaction mixture by mechanical shaking.

The XRD patterns of MNP, AMNP, EAMNP (magnetic nanoparticles modified with APTES and EPTAC), GEAMNP, and BCL-GEAMNP are shown in Fig. 4Da–e. Naked Fe_3_O_4_ had six diffraction peaks located at: 2*θ* = 30.36°, 35.79°, 43.39°, 53.66°, 57.29° and 62.92°, corresponding to the (220), (311), (400), (422), (511) and (440) crystal face of inverse spinel Fe_3_O_4_ with a face-centered cubic phase. The characteristic peaks did not change after modification with APTES, EPTAC, glutaraldehyde or lipase, which indicates that modification and immobilization do not affect the trans-cubic-spinel structure of Fe_3_O_4_ nanoparticles^41,42^. Therefore, the average size of the bare Fe_3_O_4_ nanoparticles was calculated to be 13 nm according to Debye–Scherrer equation. The modified nanoparticles, GEAMNP, aggregated in the magnetic field, indicating that modified magnetic nanoparticles immobilized with lipase may be easily separated from the reaction medium.

**Figure 5:**
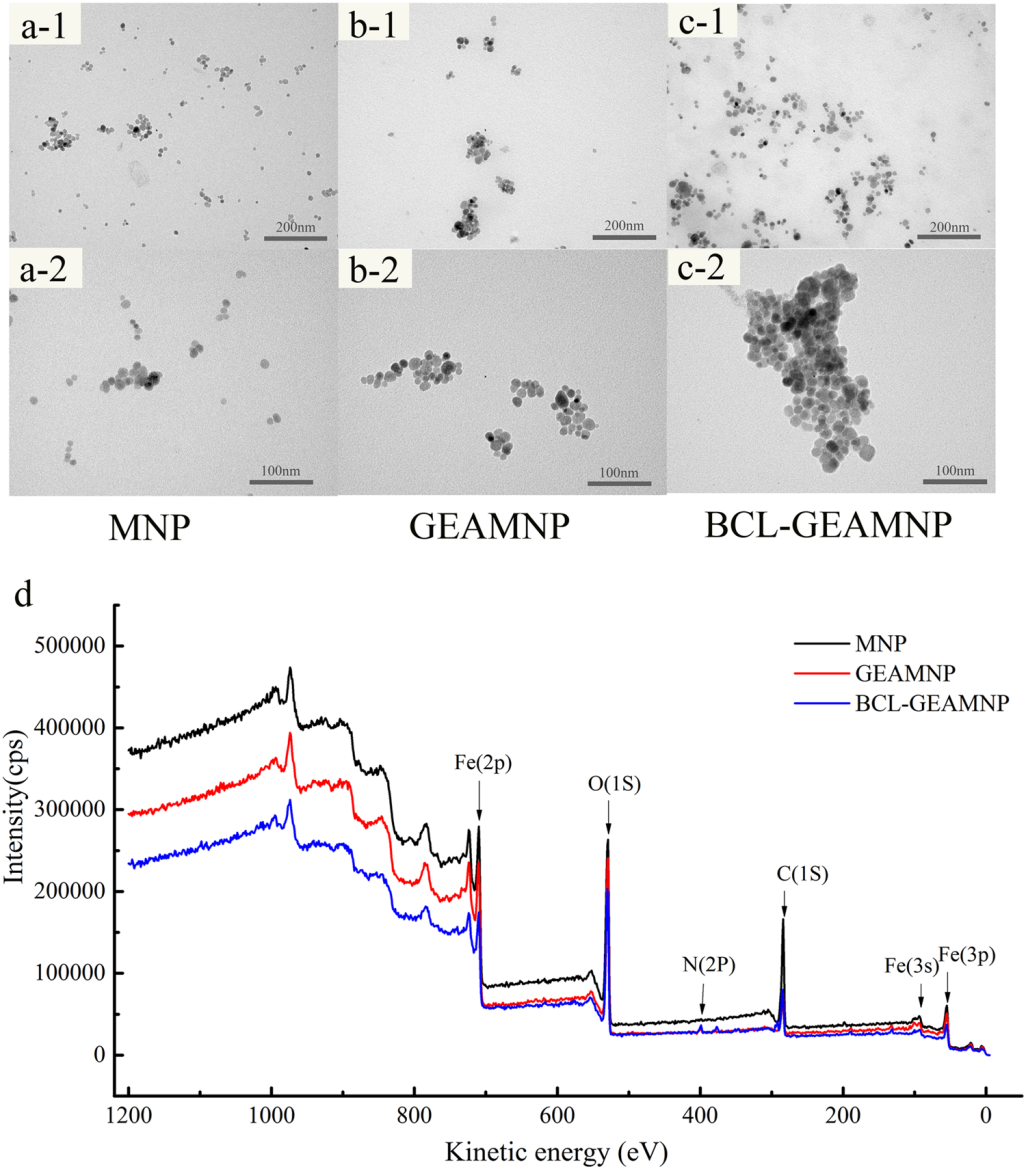
Transmission electron microscopy (TEM) images of MNP (**a**); GEAMNP (**b**) and BCL-GEAMNP (**c**); X-ray photoelectron spectroscopy (XPS) spectra of MNP, GEAMNP and BCL-GEAMNP (**d**).

The TEM images shown in Fig. 5a-1 to 5-c-2 demonstrate that the particle size of MNP (Fig. 5a-1 and a-2), GEAMNP (Fig. 5b-1 and b-2) and BCL-GEAMNP (Fig. 5c-1 and c-2) were approximately 10 to 25 nm. After modification and immobilization, particles tended to agglomerate during the process (Fig. 5b and c). The core-shell structure of GEAMNP is shown in Fig. 5b. Some extremely small particles appeared on the surface of the carriers (Fig. 5c). However, the size of the BCL molecule is very small, only 89 Å × 46 Å × 85 Å (PDB: 1YS2). It was not sure whether these small particles were protein molecules.

The XPS spectra of naked Fe_3_O_4_, modified Fe_3_O_4_, and immobilized lipase are shown in Fig. 5d. Fe_3_O_4_ only consists of Fe and O. C probably came from carbon labels added together with the sample. Thus, the main peaks of Fe_2p_ (710), C_1S_ (530) and O_1S_ (530) were present in all spectra (Fig. 5d and Tables 1, 2 and 3). After modification with organic molecules (APTES, EPTAC and glutaraldehyde), Si and N were added into the carriers (Table 2). When BCL was immobilized onto the carrier, S appeared on the surface of the carrier which was due to cysteine in the lipase (Table 3). The variation of elementary types and contents is a direct proof of successful modification and immobilization because Si only exists in APTES, N does not exist in naked Fe_3_O_4_, and S merely exists in lipase.

**Table 1:** Element information of MNP.

Peak	Position BE (eV)	Atomic Mass %	Atomic content %	Mass Content %
Fe2p	710.47	55.85	5.82	20.96
O1s	529.97	16.00	23.85	24.60
C1s	284.97	12.01	70.33	54.45

**Table 2:** Element information of GEAMNP.

Peak	Position BE (eV)	Atomic Mass %	Atomic content %	Mass Content %
Fe2p	710.31	55.87	15.52	41.26
O1s	529.76	16.00	47.90	36.48
N2p	402.00	32.07	1.03	0.69
C1s	284.97	12.01	33.91	19.39
Si2p	101.57	28.09	1.63	2.18

**Table 3:** Element information of BCL-GEAMNP.

Peak	Position BE (eV)	Atomic Mass %	Atomic content %	Mass Content %
Fe2p	710.31	55.85	11.39	33.31
O1s	529.76	16.00	45.92	38.46
N2p	399.89	32.07	2.95	2.17
C1s	285.11	12.01	38.45	24.18
Si2p	102.16	28.09	1.27	1.87
S2p	165.71	32.07	0.01	0.02

**Figure 6:**
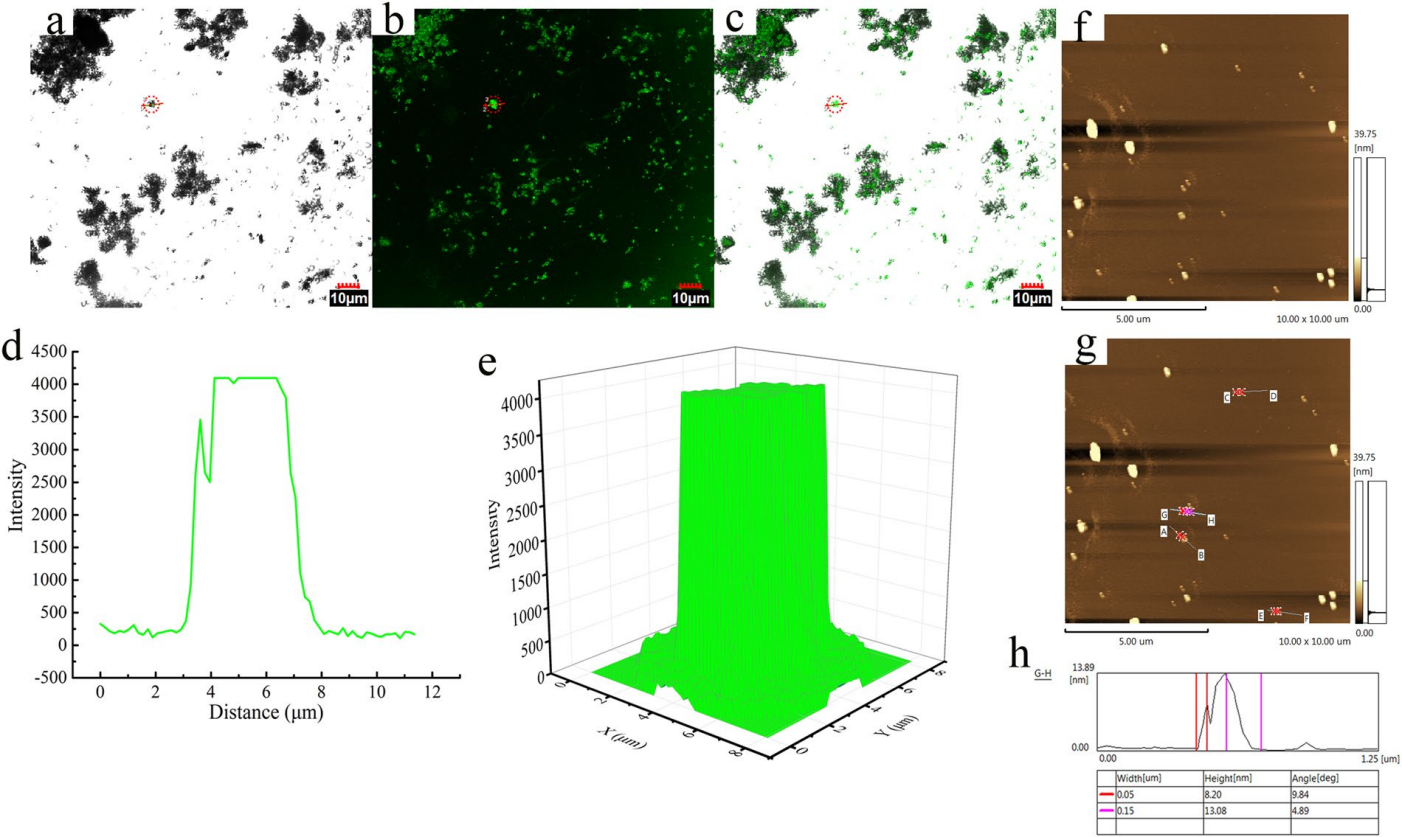
Distribution of lipase on the carrier based on confocal laser scanning microscopy (CLSM). (**a**) Mixed image of bright field and confocal fluorescence of BCL-GEAMNP. (**b**) Confocal fluorescence image of BCL-GEAMNP. (**c**) Bright field optical microscopic image of BCL-GEAMNP. (**d**) Two-dimensional fluorescence intensity distribution along the line in the CLSM image. (**e**) Three-dimensional fluorescence intensity distribution inside the circle in the CLSM image. Atomic force microscopy (AFM) images of BCL-GEAMNP: (**f**–**g**) 10 μm × 10 μm two dimensional AFM image of BCL-GEAMNP. (**h**) Particle size analysis of BCL-GEAMNP.

In order to further confirm the success of immobilization, CLSM is used to illustrate the location and distribution of the lipase on the surface of the carriers (Fig. 6a–e). CLSM provided visual evidence which directly confirmed the presence of the targeted BCL molecules on GEAMNP. The bright images were obtained using an inverted phase contrast microscope. The green signal on the image was derived from the BCL tagged with fluorescein isothiocyanate (FITC)^4^. The magnetic nanoparticles formed aggregates during synthesis, modification and immobilization. Under the bright field of CLSM, there are many large particles composed of independent small particles. All the green signals appeared on the surface of the carrier (Fig. 6a,b and c). Thus, images of bright field and confocal fluorescence for BCL-GEAMNP suggest the presence of FITC-labeled BCL on GEAMNP.

The morphology of the immobilized lipase was further characterized using AFM (Fig. 6f–h). Independent particles and aggregates are observed (Fig. 6f and h). From the results shown in Fig. 6h G–H, the sizes of the two connective independent particles are 8.20 (red) and 13.08 (purple) nm, which coincide with the sizes of the molecule and GEAMNP, respectively.

### Preparation of BCL-GEAMNP

**Figure 7:**
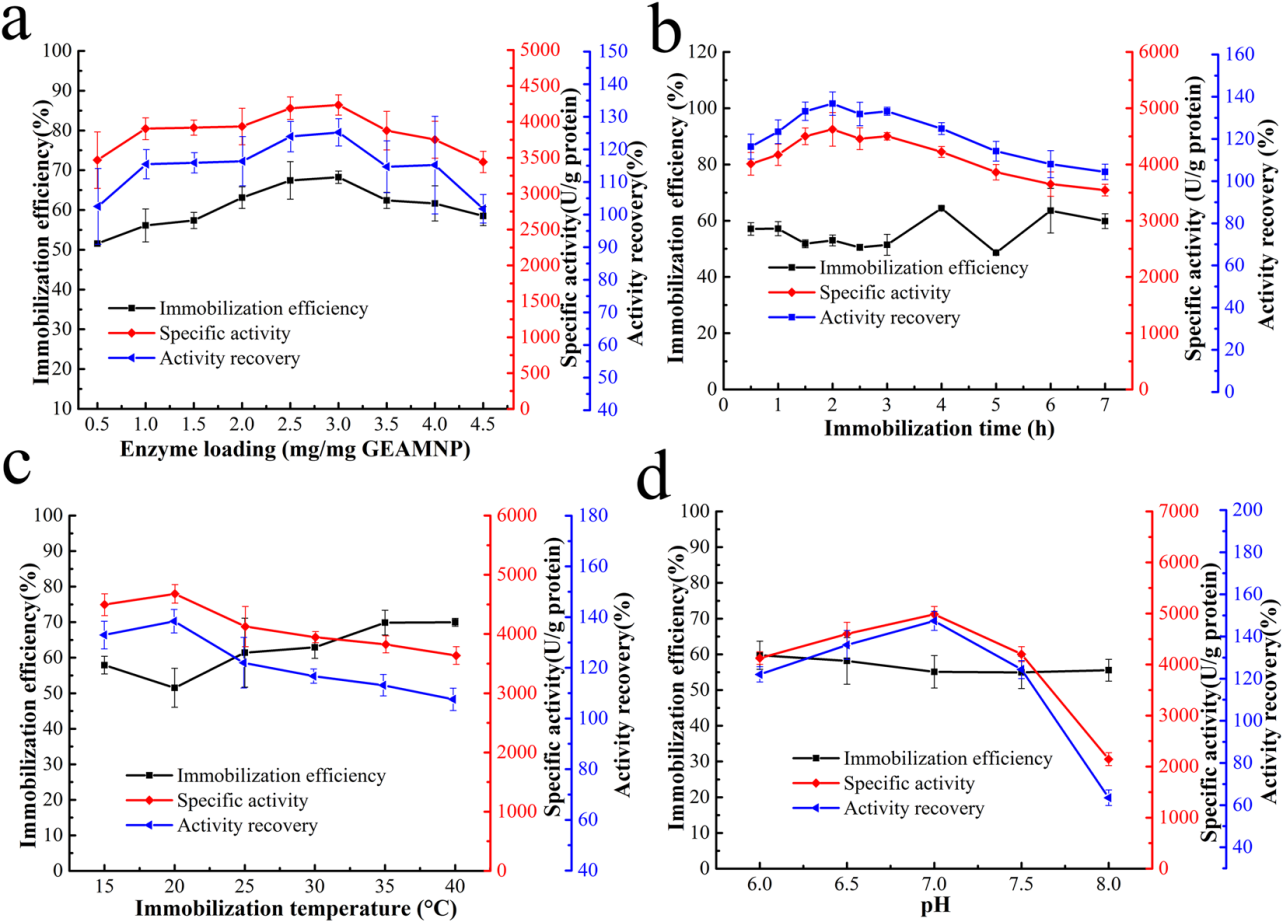
Effect of different immobilization conditions on immobilization efficiency, specific activity and activity recovery. (**a**) Effect of enzyme loading. (**b**) Effect of immobilization time. (**c**) Effect of immobilization temperature. (**d**) Effect of pH value.

As known, immobilization conditions have obvious effects on the immobilization efficiency, specific activity and activity recovery. In this study, enzyme loading, immobilization time, immobilization temperature and pH value were totally investigated. The enzyme loading ranged from 0.5–4.5 mg/mg GEAMNP, and the maximal activity recovery of 125.2% was obtained when the enzyme loading was 3 mg/mg GEAMNP (Fig. 7a). Thus, 3 mg/mg GEAMNP was selected as the appropriate enzyme loading for later study.

As shown in Fig. 7b, the specific activity and activity recovery increased in the period from 0.5 h to 2 h. The activity recovery has no obviously difference between 1.5 h to 3 h and the immobilization efficiency did not show any significant change between 0.5 h to 7 h. Therefore, 2 h (activity recovery 136.8%) was chosen as the most appropriate time for BCL immobilization.

Immobilization temperatures ranging from 15 °C to 40 °C were also investigated. The immobilization efficiency slowly increased as the immobilization temperature rose from 15 °C to 40 °C. Activity recovery reached 138.4% when the temperature was 20 °C. Beyond this temperature, the specific activity of the immobilized lipase and activity recovery began to decline (Fig. 7c). Therefore, 20 °C was set as the optimal immobilization temperature.

The pH value is an important factor for enzyme immobilization. It can be seen from Fig. 7d that a neutral environment was beneficial for BCL immobilization. The activity recovery reached its maximal value of 147.4% when the pH of the buffer was 7.0. However, the immobilization efficiency did not change markedly at different pH values. Thus, phosphate buffer was used in all immobilization processes with the highest activity recovery at pH 7.0.

Therefore, the maximal activity recovery was 147.4% under the single-factorial optimized immobilization conditions: enzyme loading 3 mg/mg GEAMNP, immobilization time 2 h, immobilization temperature 20 °C and phosphate buffer at pH 7.

### Effect of organic solvents and reaction conditions on enzymatic kinetic transesterification for biodiesel production

After immobilization and freeze-drying, BCL-GEAMNP was used to catalyze transesterification reaction between oil and methanol. In order that the immobilized lipase performed well in biodiesel preparation, organic solvents for dissolving oil and methanol were selected, and reaction parameters (lipase dosage, amount of *tert*-butanol added, molar ratio of methanol to oil, method of methanol addition, water content, reaction temperature and reaction time) were further optimized. The immobilized lipase did show high catalytic activity under the optimized conditions.

Various organic solvents (*tert*-butanol, cyclohexane, *n*-hexane, *n*-heptane, *n*-octane, isooctane, *n*-nonane, *n*-decane, *n*-undecane) were used in the transesterification system to dissolve oil and methanol (Table 4). All methanol was added in one step at the beginning of the reaction. The immiscibility of oil and methanol may have a negative effect on biodiesel production as the lipase on the carrier can be inactivated when directly contacted with a mass of insoluble methanol, because methanol can strip the necessary water surrounding the lipase. In this study, each solvent was reused for three batches to compare their reusability. Cyclohexane, *n*-hexane, *n*-heptane, *n*-octane, isooctane, *n*-nonane, *n*-decane and *n*-undecane catalytic systems performed better than *tert*-butanol system in the first batch. However, all the hydrophobic organic solution systems were unable to maintain the same high biodiesel yield in the second and third batches. Only *tert*-butanol system could. In consideration of the optimization of various reaction conditions, the performance of *tert*-butanol system could be better and was selected as the reaction medium for biodiesel preparation.

**Table 4:** The effect of different organic solvent on biodiesel yield catalyzed by BCL-GEAMNP.

Organic solvent	Reusability	Immobilization efficiency (%)	Biodiesel yield (%)
tert-Butanol	1	65.5 ± 4.5	52.0 ± 2.3
	2		49.1 ± 0.4
	3		42.0 ± 1.7
Cyclohexane	1	59.1 ± 0.8	76.2 ± 7.3
	2		38.9 ± 1.3
	3		39.7 ± 1.7
n-Hexane	1	58.9 ± 0.3	100.9 ± 8.4
	2		44.7 ± 4.8
	3		70.8 ± 3.5
n-Heptane	1	59.2 ± 2.3	76.4 ± 2.5
	2		61.2 ± 6.1
	3		71.2 ± 9.7
n-Octane	1	56.6 ± 2.9	100.1 ± 1.2
	2		48.5 ± 3.2
	3		58.1 ± 6.7
Isooctane	1	57.4 ± 3.2	86.0 ± 4.0
	2		51.3 ± 7.1
	3		65.7 ± 1.6
n-Nonane	1	56.5 ± 2.7	67.2 ± 6.8
	2		50.6 ± 9.7
	3		67.4 ± 8.6
n-Decane	1	59.2 ± 0.7	100.4 ± 8.8
	2		41.7 ± 3.9
	3		47.6 ± 2.1
n-Undecane	1	56.5 ± 1.7	83.8 ± 1.4
	2		26.4 ± 7.9
	3		32.8 ± 3.7

**Figure 8:**
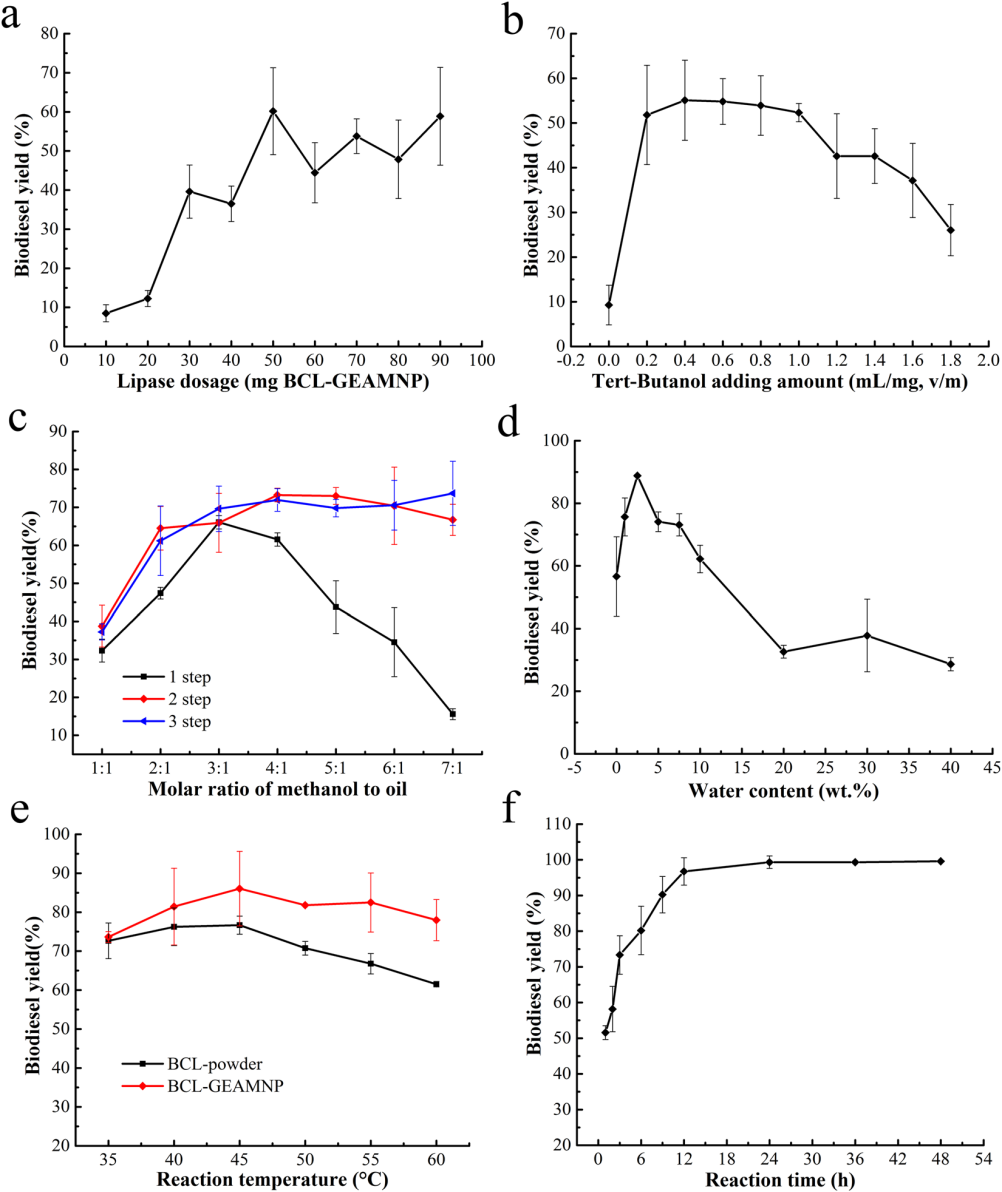
Optimization of biodiesel production. (**a**) Influence of lipase dosage. (**b**) Influence of the amount of *tert*-butanol added. (**c**) Influence of molar ratio of methanol to oil and method of methanol addition. (**d**) Influence of the amount of water added. (**e**) Influence of reaction temperature. (**f**) Influence of reaction time.

A series of reaction conditions were investigated to determine the optimum reaction conditions for biodiesel production. The effect of lipase dosage on biodiesel yield was examined first. The biodiesel yield rose gradually as the lipase dosage increased from 10 mg BCL-GEAMNP to 50 mg BCL-GEAMNP. The biodiesel yield increased to 60.2% when the lipase dosage was 50 mg BCL-GEAMNP (9.09 wt.%, based on the oil mass, g). There were no significant increases in biodiesel yield at higher lipase dosages (Fig. 8a). Therefore, the lipase dosage of 50 mg BCL-GEAMNP was selected as the best for biodiesel production.

As shown in Fig. 8b, when the amount of *tert*-butanol added changed from 0 to 0.4 mL/g oil (0.31 wt.%), the maximal biodiesel yield was 55.1%. Denaturation of the lipase caused by methanol is the main obstacle in biodiesel production^43^. Figure 8c shows the effects of different molar ratios of methanol to oil and the method of methanol addition on biodiesel production. In previous studies, methanol was added into transesterification reaction system step by step at the same time interval. In this study, the methods of methanol addition were: (1) one step: methanol was added in one step; (2) two steps: methanol was added in two steps at an interval of 4.5 h; (3) three steps: methanol was added in three steps at intervals of 3 h. The results revealed that there was no obvious difference between the two steps and three steps methods. With the two steps and three steps, the biodiesel yield was more than 70% when the molar ratio of methanol to oil was 4:1, but was only 61.5% when the one step method was used under the same reaction conditions. However, the maximal biodiesel yield could attained 66.1% at molar ratio of methanol to oil at 3:1 for one step method. Thus, the two steps method was selected as the optimal approach for biodiesel production. Additionally, the optimal molar ratio of methanol to oil was set at 4:1.

Water plays an important role in reactions and has a strong influence on the activity and stability of lipase^44^. A certain amount of water is usually needed to maintain the native conformation and catalytic activity of lipase. As depicted in Fig. 8d, biodiesel yield was only 56.6% in non-aqueous media. The highest biodiesel yield (88.8%) was obtained with 2.5 wt.% water. When the water content exceeded 10.0 wt.%, biodiesel yield decreased rapidly.

Higher temperature can accelerate the reaction rate, resulting in higher biodiesel yield. On the other hand, it can also cause denaturation of lipase. As shown in Fig. 8e, the biodiesel yield rose slowly when the reaction temperature was gradually increased from 35 °C to 45 °C, and further increment to 60 °C led to a decline in biodiesel yield. Therefore, the optimal reaction temperature was 45 °C with a biodiesel yield of 86.0%. Figure 8e also shows the difference between immobilized lipase and lipase powder. Immobilized lipase had an obvious advantage, especially at higher reaction temperatures.

The biodiesel yields at different reaction times are shown in Fig. 8f. Methanol was added in two steps at the beginning and at different times (see Supplementary Information, Table S1) during the reaction. Fatty acid methyl esters (FAMEs) increased quickly from 1 h to 9 h. Approximately 50% of the oil was transformed into methyl esters in the first 1 h, 70% in 3 h, 90% in 9 h, 96% in 12 h, and 99% in 24 h.

Thus, the biodiesel yield catalyzed by BCL-GEAMNP was 90.2 ± 5.1% with methanol added in two steps at an interval of 4.5 h and reacting 9 h, and 96.8 ± 3.8% with methanol added in two steps at an interval of 6 h and reacting 12 h, under the reaction conditions of lipase dosage 50 mg BCL-GEAMNP, amount of *tert*-butanol added 0.4 mL/g oil (0.31 wt.%), molar ratio of methanol to oil 4:1, water content 2.5 wt.%, reaction temperature 45 °C, and stirring rate at 200 rpm.

### Reusability of the immobilized BCL

**Figure 9:**
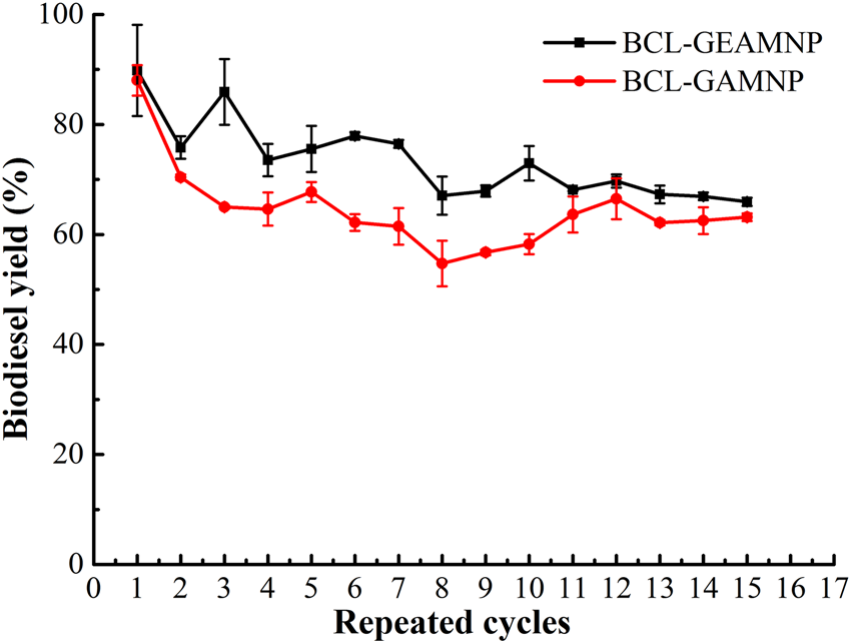
Reuse of BCL-GEAMNP (protein content 1.1%) and BCL-GAMNP (protein content 1.5%) (Reaction conditions: soybean oil 0.55 g, lipase dosage 50 mg BCL-GEAMNP, amount of *tert*-butanol added 0.4 mL/g oil (0.31 wt.%), molar ratio of methanol to oil 4:1, water content 2.5 wt.%, reaction temperature 45 °C, reaction time 9 h, time interval for methanol addition 4.5 h).

Reusability is very important for the immobilized enzymes in future practical application. To further compare the effects of the immobilization strategy combined covalent bonding with anion exchange with covalent bonding only, the immobilized lipases using different carriers such as GEAMNP and GAMNP were investigated, respectively. The two types of immobilized lipases were synthesized and operated under the same optimum immobilization conditions and transesterification reaction conditions as above-mentioned, the results are shown in Fig. 9. It can be seen that the BCL-GEAMNP (protein content 1.1%) and BCL-GAMNP (protein content 1.5%) respectively resulted in biodiesel yields of 65% and 63% after 15 cycles. Both BCL-GAMNP and BCL-GEAMNP were washed with *tert*-butanol after each batch. This manipulation was to ensure that all transesterification products from the last batch were removed from the surface of the immobilized lipases, and the immobilized lipases were further kept in fridge until the next reaction cycle. Figure 9 clearly demonstrates that BCL-GEAMNP has better operational stability in the second batch and maintains a biodiesel yield of over 75% after seven cycles, while BCL-GAMNP is lower than 70% in the third batch. These results indicate that BCL-GEAMNP was even better than BCL-GAMNP.

### Comparison between BCL-GEAMNP and other immobilized lipases

To evaluate the significance of our immobilization strategy, other recently reported immobilized BCLs were compared with BCL-GEAMNP (Table 5). You *et al*.^45^, Karimi^46^ and Jegannathan *et al*.^47^ immobilized BCL on modified attapulgite, mesoporous magnetic nanoparticles and k-carrageenan, respectively. From Table 5, it can be seen that all other immobilized BCLs needs a longer reaction time to attain high biodiesel yields. However, BCL-GEAMNP reached a high biodiesel yield of 96.8% in the first 12 h and 99.4% within 24 h, and performed much better than the others. In addition, BCL-GEAMNP also exhibited a higher reaction speed than the other commercial or synthetic immobilized lipases listed in Table 5.

**Table 5:** A comparison between the BCL-GEAMNP and the other immobilized lipases.

Lipase	Feedstock	Solvent	Enzyme concentration (% weight/oil weight)	Water content (% weight/oil weight)	Molar ratio of oil:methanol	Temperature (°C)	Reaction Time (h)	Conversion (%)	References
*B. cepacia* lipase	Jatropha oil	—	10	7	1:6.6	35	24	94.0	45
Lipase from *Burkholderia* sp. C20	Olive oil	—	40	10	1:4	40	30	92.0	68
*B. cepacia* lipase	Waste cooking oil	N-hexane	25	10	1:6	35	35	91.0	46
*B. cepacia* lipase	Palm oil	—	52.5	10	1:7	30	72	100.0	47
Novozym435	Soybean oil	Glymes	10	0.35	1.0 g soybean oil, 0.5 mL methanol	50	24	95.5	81
Lipozyme TL IM	Waste cooking oil	Polyvinyl alcohol	12	5	1:4	40	48	75.0	75
*T. lanuginosus* lipase of whole cells	Waste cooking oil	Polyvinyl alcohol	6	5	1;4	40	84	82.0	75
Novozym435	Jatropha oil	Co-solvent^a^	7.5	—	1:5	45	24	97.9	82
Lipozyme TL IM	Jatropha oil	Co-solvent^a^	7.5	—	1:5	45	24	76.5	82
Lipozyme RM IM	Jatropha oil	Co-solvent^a^	7.5	—	1:5	45	24	78.3	82
*B. cepacia* lipase	Soybean oil	tert-Butanol	9.09	2.5	1:4	45	12	96.8	This study

^a^Co-solvent: 25% t-pentanol: 75% isooctane.

## Discussion

In order to combine different advantages, such as heterofunctional immobilization strategy of being strengthened anion exchange and weakened multi-point covalent binding, easy separation, effective active sites, into one system for lipase immobilization, in our studies, we developed a novel immobilized lipase, BCL-GEAMNP.

Heterofunctional immobilization is regarded as a new method of enzyme immobilization. Of all the immobilization methods, covalent bonding has the strongest enzyme-support interaction compared with ion exchange and physical adsorption. A multipoint or multi-subunit attachment can be used to improve enzyme stability^48^. Bolivar *et al*. used a novel two steps multipoint covalent attachment to immobilize an enzyme on glyoxyl-supports. The second step of incubation at pH 10.0 promoted attachment of the enzyme through multipoint covalent immobilization and increased the stability of the immobilized enzyme 5-fold compared with the enzyme directly immobilized at pH 10.0^49^. But if some multi-interactions between the enzyme and carrier occur, distortions of enzymes will cause denaturation and losing of their activity^50^. The lipase need both rigidity and flexibility. The flexible active center of lipase may tolerate some distortions without losing activity^12^. But excessive multipoint covalent bonding will lead to the irreversible distortion of active center and the risk of losing function^6,35,51^. However, if some covalent bonds located in partial points of carrier and protein can be replaced with some weaker and reversible bonds such as ion exchange bonds or physical adsorption bonds to realize the first quick adsorption of the target protein^15^, the following irreversible multipoint covalent bonds can stabilize the target protein on the carriers. Here are some examples compared the difference between physical adsorption and covalent bonding. Awsiuk *et al*. compared physical adsorption and covalent bonding using AFM, angle-resolved XPS, and time of flight secondary ion mass spectrometry (ToF-SIMS) analysis. The AFM data suggested that protein immobilization using physical adsorption is faster than that using covalent bonding. However, conformation of the adsorbed protein changed to a flatter one compared with covalently bound molecules^52^. Holt *et al*. studied the interfacial dynamics of covalent bonding versus physical adsorption and found their difference. Three different molecules connected on the surface of silicon nanoparticles using covalent bonding present a more orderly distribution. They ascribed this difference in the interfacial properties of the two carriers to the changes in chain stretching^53^. Thus, it is interesting to combine physical adsorption and covalent bonding and determine their synergetic effects. Boros *et al*. immobilized *C. antarctica* lipase on mixed-function-grafted silica gel supports by hydrophobic adsorption together with covalent bonding^19^. Garmroodi *et al*. immobilized *Rhizomucor miehei* lipase on hetero-functionalized siliceous supports via physical adsorption together with covalent binding^54^. Both of the immobilized lipases obtained by heterofunctional immobilization methods proved to be better than the derivatives obtained from simple physical adsorption. Wang *et al*. immobilized *B. cepacia* on MNPs by physical adsorption together with covalent bonding. The immobilized lipase exhibited improved thermal stability, better tolerance to organic solvents and higher pH stability than free lipase^21^. However, the authors did not compare the immobilized lipase and derivatives obtained from a single immobilization method. Similarly, ion exchange together with covalent binding is also worthy of study. Attributed to weaker interactions, heterofunctional immobilization, anion exchange and covalent binding, can decrease inactivation of the lipase. Lipase-catalyzed biodiesel production has gained considerable attention. A short chain alcohol, such as methanol and ethanol, can replace the essential water surrounding the lipase^55^. Thus, deactivation of the biocatalyst caused by short chain alcohols is the main obstacle in biodiesel production. Therefore, it is necessary to study heterofunctional immobilization methods to retain enzyme activity and improve stability rather than using a single immobilization method.

In this study, we combined anion exchange groups (quaternary ammonium groups) and covalent groups (aldehyde groups) on the surface of the magnetic nanoparticles. It should be pointed out that this carrier had multi-point covalent binding through aldehyde group, and on the other hand, newly introduced quaternary amine group to establish and strengthen anion exchange, so as to keep the activity of the target enzyme to be immobilized as possible as it could. BCL was chosen as the biocatalyst for biodiesel production due to the results obtained in previous studies^45,56,57^.

**Figure 10:**
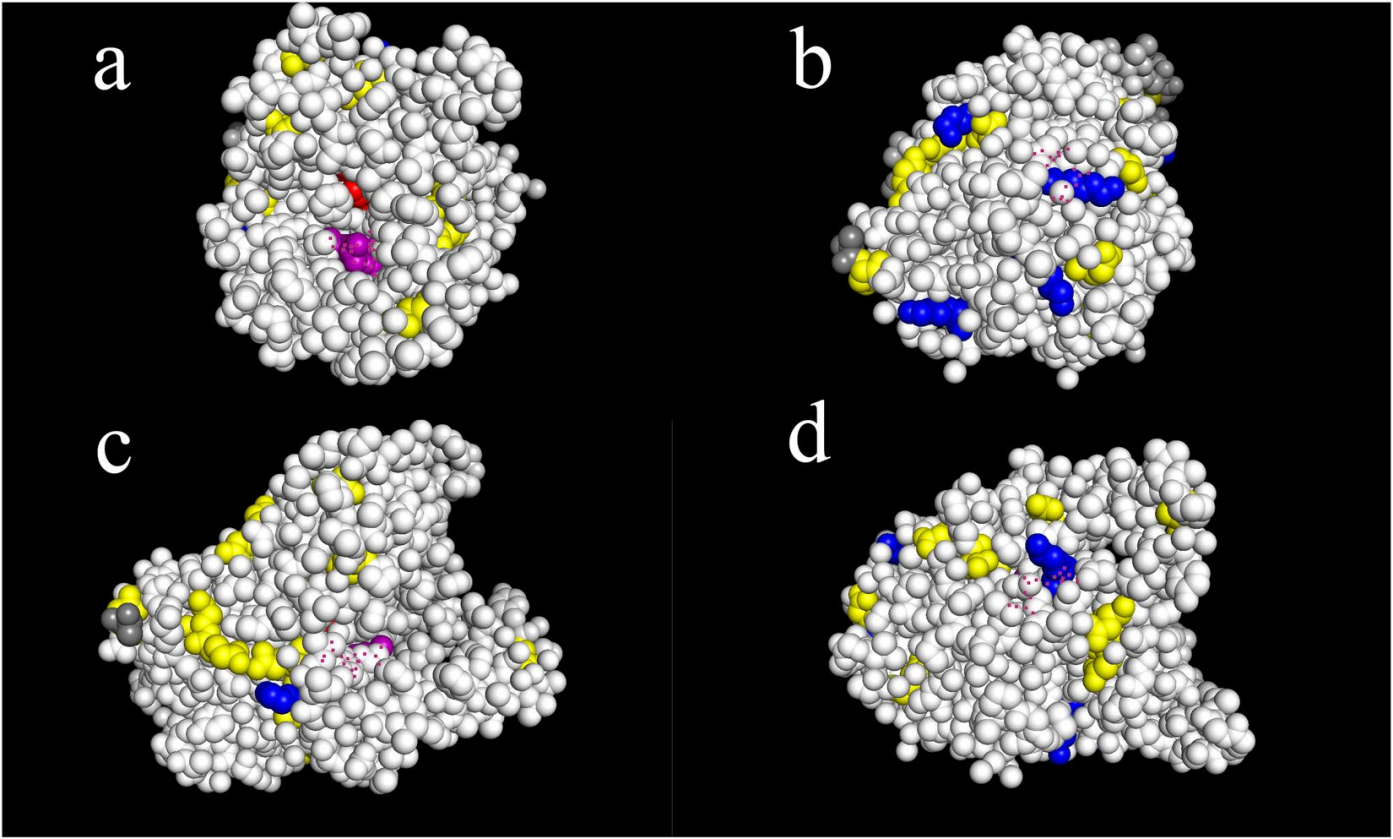
The distribution of amino groups and carboxyl groups in *Burkholderia cepacia* lipase (BCL) molecules: Lys residues (amino groups) are blue, terminal amino group is grey, Glu and Asp residues (carboxyl groups) are yellow, and the red and purple regions represent catalytic active sites and an oxyanion hole, respectively.

As shown in Table 5, many materials such as modified attapulgite, ferric silica nanocomposite, mesoporous magnetic nanoparticles and k-carrageenan have been used as carriers in BCL immobilization. Of these carriers, MNPs, which have some advantages in high protein loading or separation and recycling procedures, were selected as the immobilization carriers. The exposed amino acid residues on the BCL molecule (PDB: 1YS2) were investigated in “Supplementary Information, Figure S1”. As is known, functional groups on the surface of target protein, including amino, carboxyl, hydroxyl and sulfhydryl groups, were always used to make covalent bonds in immobilization. The percentages of these groups in decreasing order were: hydroxyl (22.9%), carboxyl (6.9%), amino (2.2%) and sulfhydryl (0.6%) (From “http://web.expasy.org/compute_pi/”). It is clear that there are sufficient functional groups to form covalent bonds. However, as discussed above, covalent bonds formation will lead to the irreversible distortion of active center and the risk of activity loss. The more covalent bonds formed, the greater this denaturation is^37,58^. There are three situations to cause loss in activity of a lipase: I. If the covalent bonds between groups of the carriers and amino acid residues near the active center formed, the access to the active center of the substrates will be hindered, and increases mass transfer resistance, leading to loss of activity; II. To be activated, the lipase needs to open its lid, then the active center can be exposed to the environment and contact with the substrate. If the covalent bonds formed on the lid of the lipase, it will also affect the formation of the open conformation of the lipase, resulting in loss in activity. III. By the way, any multiple covalent bonds formed near the active center will alter the formal configuration of the lipase, also leads to loss of activity and/or stereo-, regional-specificity. As shown in Figure S1, BCL molecule has a high density of lysine residues on the back of its catalytic center (Fig. S1a). The terminal amino group is also located on the back of the active center (Fig. 10). This type of distribution can minimize distortion of active center for BCL molecule due to covalent bonding relied on amino groups of enzyme^37^. The distributions of carboxyl groups and hydroxyl groups are uniform, with large numbers of them distributed around the catalytic site of BCL molecule (Fig. S1b and c). The forming of covalent bonds will lead to the irreversible distortion of active center. Besides, the content of hydroxyl groups is too high for covalent bonding and covalent bonding in these sites may lead to greater activity loss. As for sulfhydryl groups, reversible immobilization of the exposed nonessential thiol (SH) groups in enzyme and thiol-reactive supports have been developed under mild conditions^59,60^. However, the BCL molecule only has two cysteine residues which are engaged in the disulfide bridge (Fig. S1d). From the above analysis, the amino group in lysine was set as the main covalent bonding group (Fig. S1a and Fig. 10a). In order to further minimize the conformational change caused by excessive multipoint covalent bonding, quaternary ammonium groups were introduced into the carriers. Quaternary ammonium groups are not affected by the buffer pH compared with other amino groups and can adsorb target protein quickly. The anion exchange would happen between quaternary ammonium group of the carrier and negatively functional group like carboxyl groups of the protein. As shown in Fig. 10a and b, the distribution of aspartate and glutamate (containing carboxyl groups) was uniform. The theoretical pI of BCL molecule (PDB: 1YS2) is 5.42 (From “http://web.expasy.org/compute_pi/”) and the entire molecule exhibits electronegativity in phosphate buffers (pH = 7.0), which can lead to the anion exchange. Anion exchange existing between quaternary ammonium group of the carrier and negatively charged molecules was also reported by Belkhir *et al*., El-Boubbou *et al*. and Gomes *et al*.^61–64^. Belkhir *et al*. developed quaternary ammonium grafted polymers and used them to attract negatively charged phospholipids. Ionic bounds were successfully formed between them in their work. El-Boubbou *et al*. modified acid-prepared mesoporous silica (APMS) with quaternary ammonium ions on its external surface to immobilize organophosphorus hydrolase (OPH). El-Boubbou *et al*. and Gomes *et al*. have proven that both van der Waals force and ion exchange are playing an important role in the carrier’s interacting with OPH and subsequently improving its activity.

After obtaining GEAMNP, the immobilization conditions were optimized, and enzyme loading, immobilization time, immobilization temperature, and buffer pH are four important immobilization conditions^36,65–67^. As shown in Fig. 7, when initial enzyme loading increased, the chances of the lipase attaching to the carriers also increased, leading to an improvement in immobilization efficiency. When immobilization reached oversaturation, most of the active functional groups on the surface of the carrier were attached to lipase molecules. The enzyme loading in the present study was less than that in the studies by You *et al*.^45^, Tran *et al*.^68^, Karimi^46^, and Jegannathan *et al*.^47^ (Table 5). After optimization of initial enzyme loading, the effect of immobilization time was investigated. The immobilization time (from 0.5 h to 7 h) had no effect on immobilization efficiency, and specific activity and activity recovery also had no obvious difference (from 1.5 h to 3 h). The ion-exchange interaction firstly resulted in the lipase being adsorbed onto the surface of the carrier, then covalent bonds formed between amino groups of lipase and aldehyde groups of carrier to make the interaction stronger^36,37,40^. With regard to the effect of immobilization temperature (Fig. 7c), higher temperature accelerated the immobilization process, but was not good for maintaining the stability of BCL’s conformation^67^, especially in the reaction system with a lot of methanol. Quaternary ammonium groups have anion exchange capacity under different pH values. It is suggested that neutral pH could benefit the immobilization of BCL on heterofunctional carriers^69^.

The reaction conditions including lipase dosage, amount of *tert*-butanol added, molar ratio of methanol to oil, method of methanol addition, water content, reaction temperature and reaction time were also investigated in this study. We used optimum reaction conditions to compare the effects of BCL-GEAMNP and BCL-GAMNP. Biodiesel yield was enhanced by increasing the lipase dosage. When the lipase dosage was higher than 50 mg BCL-GEAMNP (9.09 wt.%), the biodiesel yield did not increase further, which was due to the mass transfer resistance of immobilized lipase and inactivation caused by methanol^48,54^. Organic solvents can also enhance biodiesel yield. Before optimization, different types of organic solvents were used in the reaction system to dissolve the oil and methanol. All solvents were reused three times (Table 4). As shown in Table 4, the immobilized lipase was inactivated in the hydrophobic solvent reaction systems after the first batch. This phenomenon was seen in the cyclohexane, *n*-hexane, *n*-heptane, *n*-octane, isooctane, *n*-nonane, *n*-decane, and *n*-undecane systems (Table 4). The hydrophilic organic solvent, *tert*-butanol, showed the most stable result. The result for the selection of organic solvents was also confirmed by many other studies. Li *et al*. prepared whole cell catalyzed biodiesel production in a solvent-free and *tert*-butanol system and found that the whole cell stability was significantly improved compared with that in the solvent-free system^70^. Raita *et al*. proved that the stability of immobilized lipase was enhanced by treatment with *tert*-butanol^71^. Wang *et al*. adopted *tert*-butanol as the reaction medium for biodiesel production and found that the negative effects caused by excessive methanol and glycerol attachment could be eliminated completely^72^. The immiscibility of oil, short chain alcohols and biodiesel is one of the main obstacles in biodiesel synthesis^73^. Methanol and ethanol cannot be dissolved in oil and biodiesel. When the immobilized lipase and all the liquids are mixed in the flask, lipase will undergo a conformational change and will be activated by the oil–water interface. The hydrophobic amino acids which contain the active center inside the protein are then exposed^67^. The lipases are surrounded by layers of water molecules to maintain their flexibility^39,55^. When lipase comes into contact with a high concentration of methanol, methanol can replace water on the surface of lipase and inactivate the protein^55^. *Tert*-butanol can dissolve oil, alcohol and biodiesel and was selected as the co-solvent which performed well in biodiesel production^37,67,74,75^. In the present study, the amount of *tert*-butanol added and the water content in the system were studied (Fig. 8b and d). The amount of *tert*-butanol and essential water in the microenvironment were optimum for biodiesel production. The optimum conditions were 0.4 mL/g oil (0.31 wt.%) and 2.5 wt.% water, respectively. Many studies support our results^37,67,70–72^. The method of methanol addition and molar ratio of methanol to oil were studied and the results are shown in Fig. 8c. It can be seen that the two steps and three steps methods of methanol addition performed better than the one step method, especially under conditions of high molar ratio of methanol to oil (4:1–7:1). Liu *et al*. also obtained the same results and chose the two steps method of methanol addition^69^. The reaction temperature and reaction time were also studied and the results are shown in Fig. 8e and f. The immobilized lipase, BCL-GEAMNP, performed better than BCL powder with the same protein content in the reactions (Fig. 8e) and was easier to be separated from the reaction medium. The biodiesel yield reached 96.8% in the first 12 h (Fig. 8f). These results were also compared with other recently reported immobilized lipases (Table 5). Yan *et al*. developed a novel whole cell catalyst with intracellular expression of *Thermomyces lanuginosus* lipase (TLL)^75^. This catalyst had good methanol tolerance compared with the commercial immobilized TLL, Lipozyme TL IM. Su *et al*. compared three commercial immobilized lipases, Novozym435, Lipozyme TL IM and Lipozyme RM IM, in a *tert*-pentanol co-solvent mixture with the most suitable composition of 25% *tert*-pentanol: 75% isooctane (v/v)^64^. All of these immobilized lipase catalyzed reactions required longer times to reach the highest biodiesel yields compared with the transesterification reaction catalyzed by BCL-GEAMNP. In addition, the reusability of BCL-GEAMNP and BCL-GAMNP (Fig. 9) showed that BCL-GEAMNP has higher activity and better stability, resulting in higher biodiesel yield.

Generally speaking, in this study, we successfully prepared a novel heterofunctional immobilized lipase, BCL-GEAMNP. It performed much better than the other commercial or synthetic immobilized lipases and exhibited higher activity and better stability than the single mode immobilized lipase, BCL-GAMNP. This heterofunctional strategy with strengthened ion exchange and weakened multi-point covalent binding indicates the potential wide application in the future.

## Methods

### Chemicals

Lipase from *B. cepacia*, bovine serum albumin and Coomassie brilliant blue G250 were purchased from Sigma–Aldrich (Shanghai, China). Ninhydrin, phenol, ethanol, methanol, pyridine, FeCl_3_·6H_2_O, FeSO_4_·7H_2_O, ammonium hydroxide, APTES, EPTAC, glutaraldehyde, *tert*-butanol, cyclohexane, *n*-hexane, *n*-heptane, *n*-octane, isooctane, *n*-nonane, *n*-decane and *n*-undecane were bought from Shenshi Chemical Industry (Wuhan, China). Soybean oil was obtained from a local supermarket (Wuhan, China). The physical properties and the fatty acid compositions were the same as those described by Su *et al*.^4^. Fatty acid compositions are briefly listed in Table 6, and physical properties are saponification value of 183.8 mg/g, acid value of 0.23 mg/g, and average molecular weight of 917.

**Table 6:** The fatty acid constitutions of soybean oil.

Fatty acid	Mass fraction (%)
Palmitic acid (C16:0)	11.0
Stearic acid (C18:0)	23.4
Oleinic acid (C18:1)	53.2
Linoleic acid (C18:2)	7.8
Linolenic acid (C18:3)	4.0

### Synthesis of GEAMNP

MNPs and AMNPs were prepared according to the method reported by Jin *et al*.^39^, and Xie and Ma^36^. EPTAC and glutaraldehyde were added to the surface of the carriers step by step. First, AMNP and EPTAC were dispersed in an appropriate amount of water under rotation at 200 rpm and 60 °C for 24 h. After that, the MNPs with quaternary ammonium groups (EAMNPs) were separated under an external magnetic field and washed four times with deionized water. Then, 1 g EAMNPs were dispersed in 80 mL ethanol and 20 mL glutaraldehyde (25%) at 30 °C for 24 h at a speed of 200 rpm. The final particles, modified with quaternary ammonium groups and aldehyde groups, were named GEAMNPs, washed in ethanol four times, dispersed in water, and stored at 4 °C for further use.

### Quantitative evaluations of surface-concentrated amino groups on the carriers

The concentrations of amino groups on the surface of the carriers were measured according to the method described by Mori *et al*.^76^.

### Lipase immobilization

BCL molecules were conjugated with the carriers by covalent bonding and anion exchange. The GEAMNPs were dispersed in phosphate buffer (pH value ranging from 6.0 to 8.0) with BCL. After immobilization, the immobilized lipases were washed with the same buffer. All solutions were collected to obtain the residual protein content in the supernatant using the Bradford method with bovine serum albumin as the standard protein^77^. The immobilized lipases, BCL-GEAMNPs were dried in a vacuum dryer and stored at 4 °C for later use.

During optimization of the immobilization conditions, the effects of enzyme loading (0.5 mg PS/mg GEAMNP to 4.5 mg PS/mg GEAMNP), immobilization time (1–6 h), immobilization temperature (15 °C–40 °C) and pH value (pH 6–8) on the immobilization efficiency, specific activity and activity recovery of the immobilized lipase were investigated.

### Characterization

The nanoparticles modified with APTES, EPTAC, glutaraldehyde and immobilized with BCL were characterized using an X-ray diffractometer (PAN Analytical B.V., Almelo, Netherlands) with a Cu Kα radiation source in the 2θ range of 20° to 90°.

The FT-IR spectra were obtained in transmission mode by Fourier transform infrared spectroscopy (Bruker, VERTEX 70, Karlsruhe, Germany) using the KBr pellet technique^37^.

XPS measurements were recorded on a monochromated Al Kα photoelectron spectrometer^78,79^.

One drop of the sample suspension was placed in the center of the carbon-coated grid to visualize the dimensions and morphological details of the MNP, modified MNP and immobilized lipase using transmission electron microscopy (TEM, H-7000FA, Hitachi, Tokyo, Japan).

Structural characterization of the immobilized lipase was performed using AFM, NanoScope III A (Shimadzu, Kyoto, Japan). The sample was carried out in tapping mode. The particles were dispersed in ethanol and dropped onto a Si/SiO_2_ wafer.

The modified MNPs coupled with lipases were analyzed by CLSM (Olympus FV1000, Tokyo, Japan). The detailed methods were described by Su *et al*.^4^.

The magnetic properties were measured using a superconducting quantum interference device magnetometer (SQUID, Quantum Design, San Diago, USA)^37^.

### Enzyme assays

The transesterification reaction between soybean oil and methanol was used to measure the enzyme activity of the free lipase and immobilized lipase. One unit (U) of enzyme activity was defined as the amount of enzyme which produced 1 μmol FAME in 1 min under the assay conditions. The reaction system consisted of the correct amount of BCL-GEAMNP, 550 mg soybean oil, 0.55 mL *tert*-butanol, 5 μL deionized water, 0.102 mL methanol and was shaken for 9 h at 45 °C. The protein content of BCL “Amano” SD (PS) was 0.74%. The immobilization efficiency (%), activity recovery (%) and specific activity (U/g protein) were calculated via equations (1), (2), and (3)^67^.

1 $${\rm{Immobilization}}\,{\rm{efficiency}}\,( \% )=\frac{{\rm{immobilized}}\,{\rm{lipase}}}{{\rm{total}}\,{\rm{loading}}\,{\rm{protein}}}\times \mathrm{100} \% $$

2 $${\rm{Activity}}\,{\rm{recovery}}\,( \% )=\frac{{\rm{activity}}\,{\rm{of}}\,{\rm{immobilized}}\,{\rm{lipase}}}{{\rm{total}}\,{\rm{activity}}\,{\rm{of}}\,{\rm{free}}\,{\rm{lipase}}}\times \mathrm{100} \% $$

3 $${\rm{Specific}}\,{\rm{activity}}\,({\rm{U}}{\rm{/}}{\rm{g}}\,\mathrm{protein})=\frac{{\rm{initial}}\,{\rm{activity}}}{{\rm{protein}}\,{\rm{content}}\,{\rm{of}}\,{\rm{immobilized}}\,{\rm{lipase}}}$$

### Biodiesel production and gas chromatography analysis

Biodiesel production and GC analysis methods were described in our previous work^67,69,80^. Soybean oil (550 mg) and different contents of methanol, *tert*-butanol, water and BCL-GEAMNP were placed in a 50 mL flask in a shaking bed with a stirring rate of 200 rpm. When the reaction was over, 400 μL of the sample was collected from the reaction mixture and centrifuged at 12,000 rpm for 5 min to obtain 200 μL supernatant. A 10 μL sample was removed from the 200 μL supernatant, and then mixed with 300 μL of internal standard solution (1.0 mg/mL heptadecanoic acid methyl ester, hexane as solvent) and 290 μL hexane. 1.0 μL of the mixed sample was injected into the GC-9790 gas chromatograph (Fuli Analytical Instrument Co., Ltd., Wenlin, China). The column was an Agilent INNOWAX capillary column (30 m × 0.25 mm i.d. × 0.25 μm, J&W Scientific, Folsom, CA, USA). The initial temperature was 180 °C, increased to 230 °C at a rate of 3 °C/min and maintained for 2–3 min. The temperatures of the injector and detector were kept at 240 °C and 280 °C, respectively. The biodiesel yield (%) was defined as the conversion of soybean oil and calculated as the total FAME content in the conversion oil sample using equations (4), (5) and (6)^67,69,80^.

4 $${W}_{e}=\frac{{A}_{{sample}}{f}_{0}}{{A}_{{int}{e}{r}{n}{a}{l}}}{W}_{{int}{e}{r}{n}{a}{l}}$$

5 $${f}_{0}=\frac{{W}_{{s}{\tan }{d}{a}{r}{d}}A{^{\prime} }_{{int}{e}{r}{n}{a}{l}}}{W{^{\prime} }_{{internal}}{A}_{{standard}}}$$

6 $${\rm{Biodiesel}}\,{\rm{yield}}\,( \% )=\frac{{W}_{e}}{{W}_{t}}\times \mathrm{100} \% $$

where *A*_*sample*_ is the peak area of FAME in the sample, *f*_0_ is the response factor, *A*_*internal*_ and *A*′_*internal*_ are the peak area of the internal standard, and *W*_*internal*_ and *W*′_*internal*_ are the weight of the internal standard. *W*_*standard*_ is the weight of standard substance of FAME, and *A*_*standard*_ is the peak area of standard substance of FAME. *W*_*e*_ is the experimental value of all FAMEs tested with the GC, and *W*_*t*_ is the theoretical value of all FAMEs.

## Electronic supplementary material

Supplementary Information
