# Supplementary material for: Burkholderia cepacia lipase immobilized on heterofunctional magnetic nanoparticles and its application in biodiesel synthesis
Source: Sci Rep. 2017 Nov 28;7:16473. doi: 10.1038/s41598-017-16626-5 (PMC5705719; doi:10.1038/s41598-017-16626-5)
Supplement: Supplementary file 1 — Supplementary Information [file 41598_2017_16626_MOESM1_ESM.pdf]

Supplementary Information

Burkholderia cepacia lipase immobilized on heterofunctional magnetic nanoparticles and its application in biodiesel synthesis

Kai Li[a], Yanli Fan[a], Yaojia He[a], Leping Zeng[a], Xiaotao Han[b], Yunjun Yan[a*]

[a] Key Laboratory of Molecular Biophysics of the Ministry of Education, College of Life Science and Technology, Huazhong University of Science and Technology, Wuhan 430074, China

[b] Wuhan National High Magnetic Field Center, Huazhong University of Science and Technology, Wuhan 430074, P. R. China

*Corresponding author. Tel/Fax: +86-27-87792213;

E-mail: yanyunjun@hust.edu.cn

Results

Kinetic parameters of immobilized BCL

Figure S3 a and b showed the effect of reaction time on biodiesel yield and specific activity of immobilized lipase, BCL-GEAMNP. The reaction was tested under the optimized conditions. When increasing the content of oil from 0.55 g to 2.75 g in transesterification system while the lipase dosage remain unchanged, the reaction time of transforming all of the triglyceride to fatty acid methyl ester changed from 24 h to 84 h. Figure S3 c showed the Lineweaver – Burk plot of all the data when all of the system reacted for 2 h, 3 h, 6 h and 9 h. The reaction rates were monitored at FAME concentration at different initial oil content. The average K_m value is 1.4 mmoL. The V_{max} values at 2 h, 3 h, 6 h, 9 h are 14.7, 11.3, 7.2 and 5.8 mmoL/min*g protein, respectively.

Methods

Determination of K_m and V_{max} values

Kinetic parameters were determined in a mixture (50 mg BCL-GEAMNP, amount of tert-butanol added 0.4 mL/g oil (0.31 wt.%), molar ratio of methanol to oil 4:1, water content 2.5 wt.%) at 45 ℃ and 200 rpm for different quality of oil (0.55 to 2.75 g), the reaction time was tested at 2 h, 3 h, 6 h and 9 h, respectively. K_m and V_{max} were calculated from the Lineweaver-Burk equation using computer linear regression calculations (Microsoft excel 2015). The data represent the average of all statistically relevant data with a standard deviation of less than 10%.

Supplementary Figure Captions

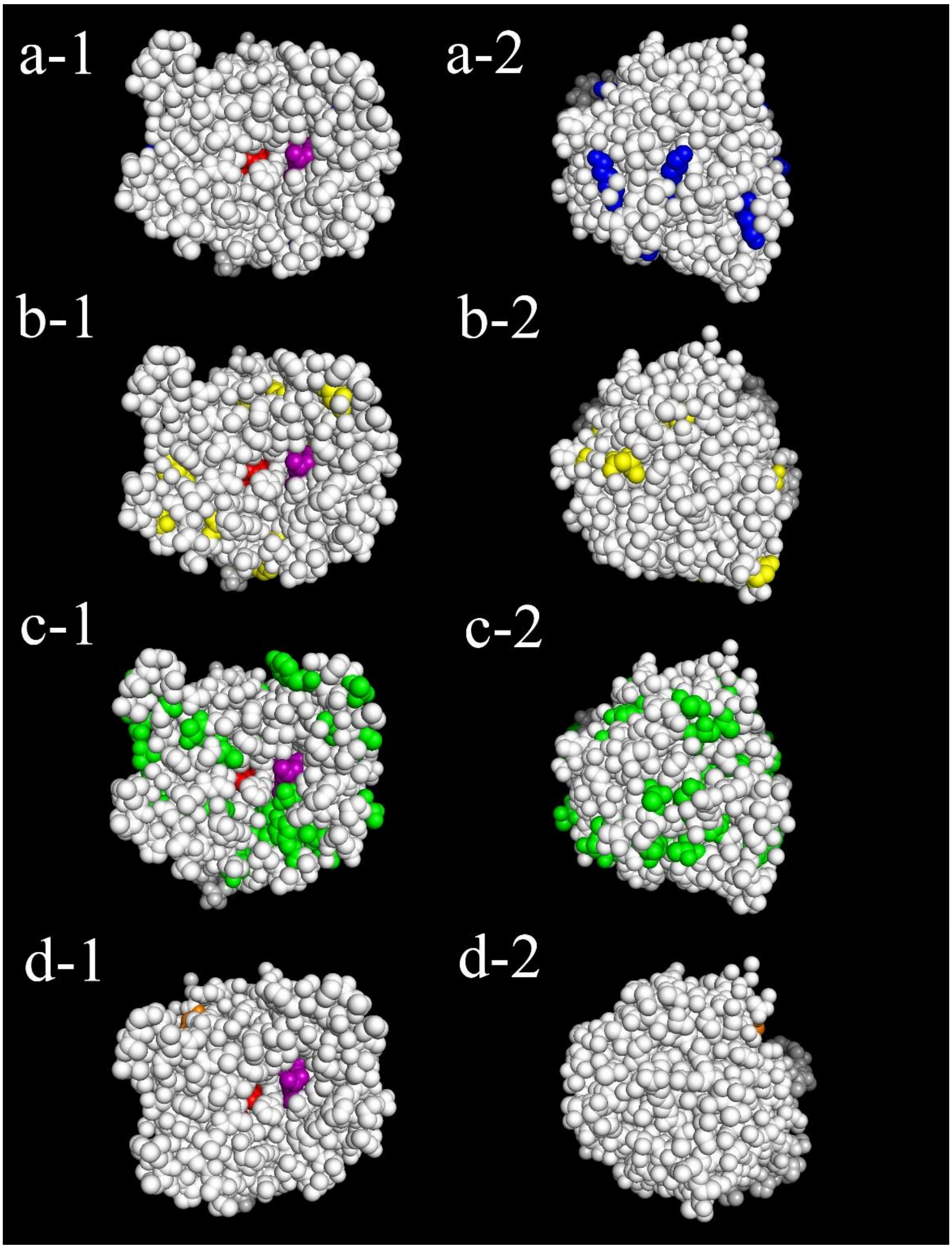

Figure S1. The distribution of amino acid residues in *Burkholderia cepacia* lipase (BCL) molecules: (a) amino group; (b) carboxyl group; (c) hydroxyl group; (d) sulfhydryl group (Lys residues are blue (a), Glu and Asp residues were yellow (b), Ser, Tyr and Thr are green (c) and Cys residues are orange (d) while red and purple regions (a-d) represent catalytic active sites and oxyanion hole, respectively).

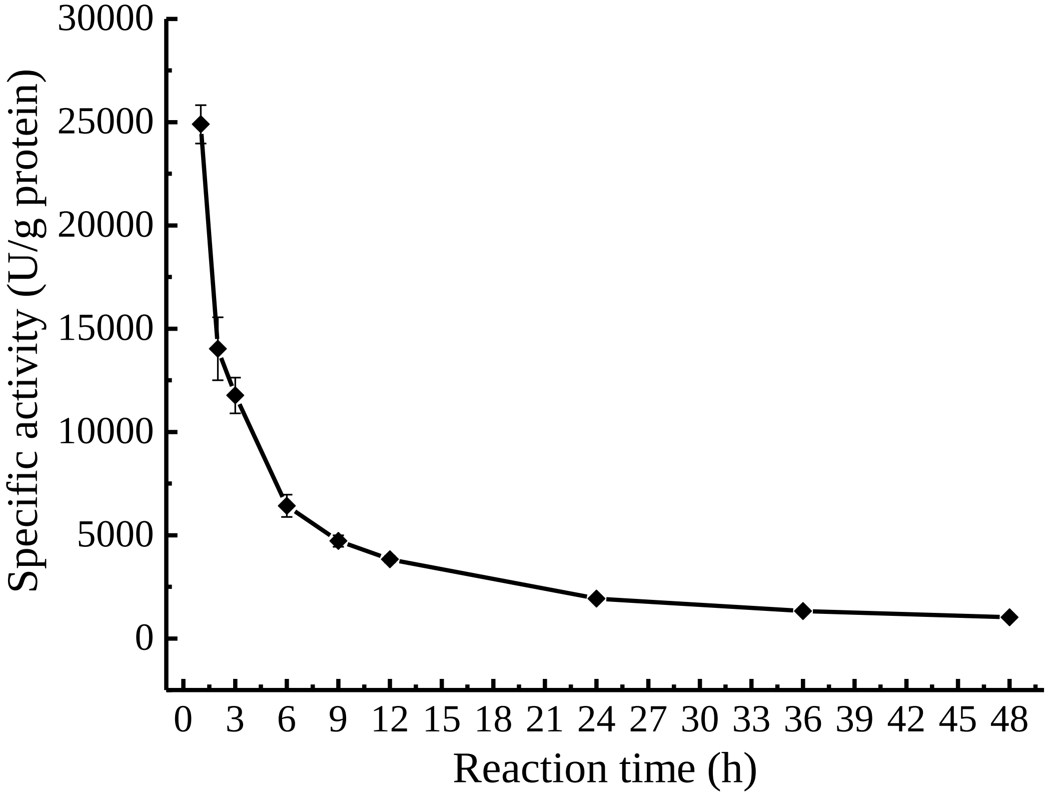

Figure S2. The effect of reaction time on specific activity of BCL-GEAMNP (Protein content 1.1%; Reaction conditions: soybean oil 0.55g, lipase dosage 50 mg BCL-GEAMNP, amount of *tert*-butanol added 0.4 mL/g oil (0.31 wt.%), molar ratio of methanol to oil 4:1, methanol added in two steps, water content 2.5 wt.%, reaction temperature 45 ℃.)

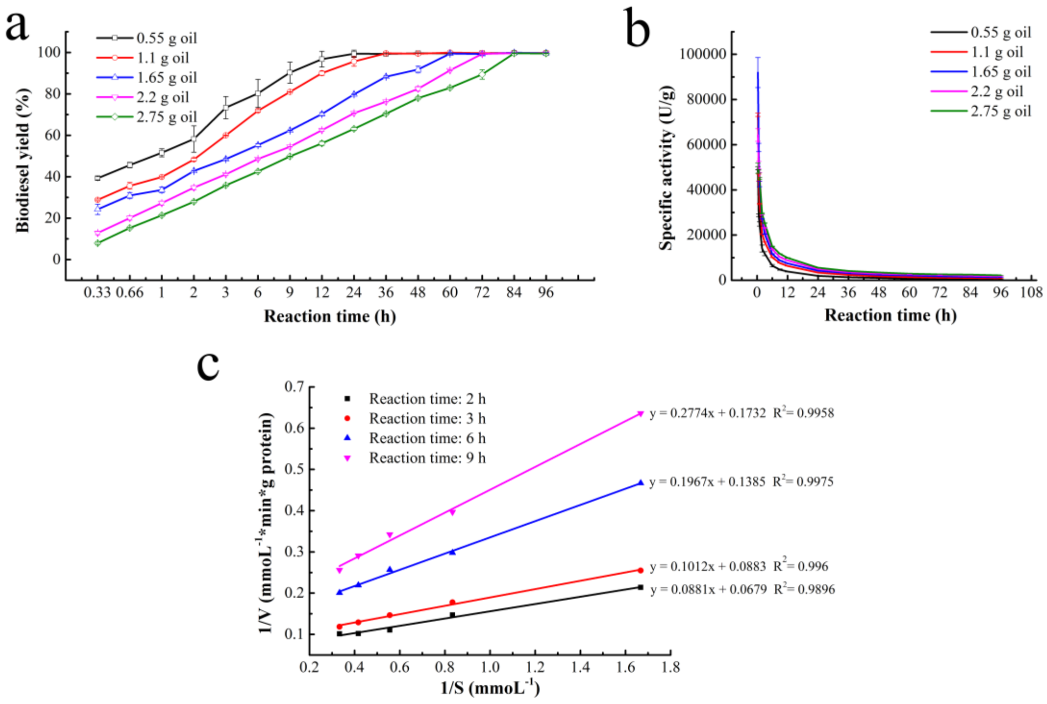

Figure S3. Kinetic analysis: the relationship between reaction time and biodiesel yield (a) and specific activity (b); Lineweaver Burk plot showing the reverse of d[S]/dt (1/V) with respect to the inverse of the initial amount of substrate (1/S) (c)

Table S1. The effect of reaction time and methanol addition time on biodiesel yield and specific activity of BCL-GEAMNP

Reaction time/h	Methanol addition time/h (the second step)	Biodiesel yield /%	Specific activity (U/g protein)
1	0.5	51.6±1.9	24896.1±925.2
2	1	58.2±6.4	14030.5±1765.1
3	1.5	73.3±5.4	11769.0±1669.3
6	3	80.2±6.8	6427.2±538.6
9	4.5	90.2±5.1	4716.2±272.0
12	6	96.8±3.8	3828.4±158.1
24	12	99.4±1.7	1930.9±175.0
36	18	89.7±5.9	1064.8±158.9
48	24	83.0±2.5	831.7±25.4
36	12	99.3±0.1	1326.8±1.3
48	12	99.6±0.2	1026.5±1.8
